# 

**Published:** 1904-03

**Authors:** 


### BOOKS RECEIVED.

Infant Feeding in its Relation to Health and Disease. A Modern Book on all Methods of Feeding. For Students, Practitioners, and Nurses. By Louis Fischer, M. D., Visiting Physician to the Willard Parker and Riverside Hospitals, of New York City; Attending Physician to the Children's Service of the New York German Poliklinik. Third Edition, Thoroughly Revised and Largely Re-written. Containing fifty-four illustrations, 357 octave pages. F. A. Davis Company, Philadelphia. 1904. (Price, $1.50 net.)

Juler's Ophthalmology. Third edition. Revised and enlarged. A Handbook of Ophthalmic Science and Practice. For Students and Practitioners. By Henry E. Juler, F. R. C. S., Ophthalmic Surgeon to St. Mary's Hospital; Surgeon to the Royal Westminster Ophthalmic Hospital, London. Octavo, 733 pages, with 190 illustrations and twenty-five full-page plates in colors and black. Lea Brothers & Co., Philadelphia and New York. 1904. (Cloth, $5.25 net.)

The American Year-Book of Medicine and Surgery for 1904. A Yearly Digest of Scientific Progress and Authoritative Opinions in all branches of Medicine and Surgery, drawn from journals, monographs, and textbooks of the leading American and foreign authors and investigators. Arranged, with critical editorial comments, by eminent American specialists, under the editorial charge of George M. Gould, A. M., M. D. In two volumes. Volume I, including *General Medicine.* Octavo, 673 pages, fully illustrated; Volume II, *General Surgery.* Octavo, 680 pages, fully illustrated. Philadelphia, New York, London: W. B. Saunders & Co. 1904. (Per volume: Cloth, $3.00 net; half morocco, $3.75 net.)

Diseases of the Eye. By L. Webster Fox, A. M., M. D., Professor of Ophthalmology in the Medico-Chirurgical College of Philadelphia; Ophthalmic Surgeon in the Medico-Chirurgical Hospital. Octavo, pp. 603, with five colored plates and 296 illustrations in the text. New York and London: D. Appleton & Co. 1904. (Price, $4.00.)

A Manual of General Pathology. For Students. By Sidney Martin, M. D., F. R. S., F. R. C. P., Professor of Pathology at University College; Physician to University College Hospital, London. Octavo, pp. 521. Illustrated. Philadelphia: P. Blakiston's Son & Co. 1904. (Price, $4.00.)

Mother and Daughter. Advice to the Maiden, Wife and Mother. By C. A. Button, M. D., Holland, N. Y., pp. 153. Holland Medical Company, Holland, N. Y. (Price, $1.00.)

Howe's Handbook of Parliamentary Usage. Arranged for the instant use of legislative and mass meetings, clubs and fraternal orders, teachers, students, etc. By Frank William Howe. New York: Hinds & Noble. (Price, 50 cents.)

Lectures on Diseases of the Nervous System. Second series. Subjective sensations on light and sound, abiotrophy and other lectures. By Sir William R. Gowers, M. D., F. R. C. P., F. R. S., Hononary Fellow Royal College of Physicians, Ireland, etc. Small 8 vo, pp. 250. Philadelphia: P. Blakiston's Son & Co. 1904.

Report of the Commissioner of Education for the year 1902. Volume 2. Washington: Government Printing Office. 1903.

Biographic Clinics. Volume II. The Origin of the Ill Health of George Eliot, George Henry Lewes, Wagner, Parkman, Jane Welch Carlyle, Spencer, Whittier, Margaret Fuller Ossoli and Nietzsche. By George M. Gould, M. D., editor of American Medicine. Duodecimo, pp. 392. Philadelphia: P. Blakiston's Son & Co. 1904.

The Complete Medical Pocket-Formulary and Physician's Vade-Mecum. Containing upwards of 2,500 prescriptions, collected from the practice of physicians and surgeons of experience, American and foreign. Also a special list of new drugs, with dosage, etc. By J. C. Wilson, A. M., M. D., physician to the German Hospital, Philadelphia. Third revised edition. Philadelphia: J. B. Lippincott Company. (Price, $1.75, third indexed, $2.)

International Clinics. A Quarterly of Illustrated Clinical Lectures and especially prepared original articles on Treatment, Medicine, Surgery, Neurology, Pediatrics, Obstetrics, Gynecology, Orthopedics, Pathology, Dermatology, Ophthalmology, Otology, Rhinology, Laryngology, Hygiene and other topics of interest to students and practitioners, by leading members of the medical profession throughout the world. Edited by A. O. J. Kelly, A. M., M. D., Philadelphia. Volume IV. Thirteenth series. 1904. Philadelphia: J. B. Lippincott Company. 1903. Cloth, $2.00.)

The practical Medicine Series of Year Books. Ten volumes. Issued under the general editorial charge of Gustavus P. Head, M. D., Professor of Laryngology and Rhinology in the Chicago Post-Graduate Medical School. Volume II. General Surgery. Edited by John B. Murphy, M. D., Professor of Surgery, Northwestern University Medical School. Duodecimo, pp. 566. Chicago: The Year Book Publishers. 1903. (Price, $1.50; entire series, $5.50, payable in advance.)

The Worth of Words. By Dr. Raley Husted Bell. With an introduction by Dr. William Colby Cooper. 12 mo, pp. 332. Third edition revised and enlarged. New York: Hinds & Noble. 1904. (Price, $1.25.)

Transactions of the American Dermatological Association. Twenty-seventh annual meeting held at Washington, D. C., May 12-14, 1903. Charles J. White, M. D., secretary. New York: The Grafton Press.

Transactions of the New York Academy of Medicine. Semi-Centennial Celebration, 1896-1901. 1896-1901, including the Semi-Centennial Celebration Addresses, January 29, 1897, and List of Papers read at Stated Meetings of the Academy. Printed for the Academy, New York, 1903.

Preventive Medicine. Two Prize Essays: The General Principles of Preventive Medicine, by W. Wayne Babcock, M. D.; the Medical Inspection of Schools, a Problem in Preventive Medicine, by Lewis S. Somers, M. D. Published by The Maltine Company, Brooklyn. 1903.

